# Conversion of Cellulose and Lignin Residues into Transparent UV-Blocking Composite Films

**DOI:** 10.3390/molecules27051637

**Published:** 2022-03-01

**Authors:** Fan Yang, Lu Xu, Guodong Dai, Lin Luo, Kaifeng Yang, Churui Huang, Dong Tian, Fei Shen

**Affiliations:** 1 Chengdu Campus, Institute of Ecological and Environmental Sciences, Sichuan Agricultural University, Chengdu 611130, China; sc_yang_fan_123@163.com (F.Y.); xulu81673422@163.com (L.X.); daiguodong0728@163.com (G.D.); lalalaluoluo@163.com (L.L.); kevinyang0913@outlook.com (K.Y.); johnson_814@163.com (C.H.); 2 Wenjiang Campus, College of Chemistry and Life Science, Chengdu Normal University, Chengdu 611130, China; 3 Sichuan Provincial Key Laboratory for Structural Optimization and Application of Functional Molecules, Wenjiang Campus, Chengdu Normal University, Chengdu 611130, China

**Keywords:** cellulose residue, lignin residue, UV-blocking, composite film, enzymatic hydrolysis

## Abstract

The valorization of cellulose and lignin residues in an integrated biorefinery is of great significance to improve the overall economics but has been challenged by their structural recalcitrance, especially for lignin residue. In this work, a facile chemical conversion route to fabricating functional UV-blocking cellulose/lignin composite films through a facile dissolution–regeneration process using these biomass residues was proposed. Three representative lignin residues, i.e., aspen and poplar wood lignin, and corn stover (CS) lignin were assessed for their feasibility for the film fabrication. The UV-blocking performance of the composite films were comparatively investigated. Results showed that all these three lignin residues could enhance the UV-blocking property of the composite films, corresponding to the reduction in the optical energy band gap from 4.31 to 3.72 eV, while poplar lignin had a considerable content of chromophores and showed the best UV-blocking enhancement among these three assessing lignins. The enhancement of UV-blocking property was achieved without compromising the visible-light transparency, mechanical strength and thermal stability of the composite films even at 4% lignin loading. This work showed the high promise of integrating biomass residue conversion into lignocellulose biorefinery for a multi-production purpose.

## 1. Introduction

The current multiple-product integrated biorefinery concept and demonstrating plant has been using lignocellulosic biomass as the starting material due to its abundance in nature, carbon-neutral status and renewable advantages [1]. It is the most promising and perhaps the only alternative to traditional fossil resources [2,3]. Unlike the well-developed oil refinery that could co-produce a wide range of platform chemicals, material precursors and energy products to achieve its complete utilization, the new-born lignocellulose biorefinery confronts tremendous technical and economic challenges to achieve such a goal. These challenges are mainly caused by the biomass recalcitrance of lignocellulose, which exhibits complex chemical compositions and entangled networks with covalent and hydrogen bonds [4]. In the traditional oil refinery, the residue named as bitumen could be well used for construction to enhance its value, while for the lignocellulose biorefinery, the residues are usually burned for heat generation [5,6]. Therefore, exploring new technical routes to realize the valorization of these biomass residues is of great significance to improve the overall economics of the biorefinery.

Despite the fast development of pursuing cellulosic ethanol from lignocellulose at both bench and industrial scale, it has still been challenging to achieve 100% cellulose conversion to glucose. In this process, a chemical or physic-chemical pretreatment step is required to open up the recalcitrant structure of lignocellulose while enhancing cellulose accessibility to hydrolytic enzymes [7]. Cellulose residue is produced at the end of the hydrolysis, while lignin residue could be produced though chemical solvent extraction during the pretreatment or co-produced in the enzymatic hydrolysis section [8]. It has been reported that the high crystalline cellulose is more recalcitrant to enzymatic hydrolysis than the amorphous one [9,10]. Further enhancing the glucose conversion requires prolonging the hydrolysis time or increasing enzyme usage, despite the intractable marginal effect. Unlike cellulose residue that has a well-defined linear macromolecular structure, lignin residue usually exhibits a random and branched three-dimensional structure that is derived from three phenylpropane units (monolignols), namely, guaiacyl (G, coniferyl alcohol), syringyl (S, sinapyl alcohol) and *p*-hydroxyphenyl (H, *p*-coumaryl alcohol). Lignin structure and composition is very dependent on biomass types and the pretreatment method selected. These disadvantages add extra technical challenge to its high-value conversion [11].

To valorize cellulose and lignin residues in the integrated biorefinery, explorations including nanocellulose production, lignin nanoparticles formation, fertilizer conversion, antioxidant production, pyrolysis and hydrothermal liquefaction upgrading, etc., have been reported [11,12,13,14,15,16]. Apart from these conversion routes, fabricating cellulose residue into film material is also a promising way since the well-developed cellulose film material platform showed tremendous applications in various fields [17]. Additionally, plastic films, mainly polyethylene films that are produced through thermal blowing, have been widely used for packaging for a long time. The improper disposal of this consumed synthetic polymer packaging films consequently caused serious environmental concerns such as marine microplastics pollution. It was anticipated that the fully biodegradable cellulose films could be a promising alternative to those synthetic polymer packaging films to alleviate plastic pollution. It should also be noted that the cellulose degree of polymerization would be significantly decreased after enzymatic hydrolysis/pretreatment, which would ease its dissolution process, thus facilitating the subsequent film-casting process [18]. To endow a film material with additional functionality such as UV-blocking property, metal oxides including titanium dioxide and zinc oxide have usually been introduced. However, this would cause some environmental concerns as well as the potential polymer macromolecular chain cleavage catalyzed by those metal oxides due to their intrinsic photocatalytic activity [19,20]. Considering that lignin residues are abundant in phenolic forms, they might be good alternatives to those metal oxides for the fabrication of cellulose composite films with UV-blocking property.

In this work, cellulose and lignin residues generated in the bioethanol refinery were fabricated into composite films using a facile solution-casting method. It was proposed that cellulose residue with a lowered degree of polymerization obtained after enzymatic hydrolysis was feasible for cellulose film preparation, while lignin residue could be used as the functional additive to enhance its UV-blocking performance. Three representative lignin residues, i.e., aspen and poplar wood lignin, and corn stover (CS) lignin were assessed for their technical feasibility for the film preparation. UV-blocking performance and the general properties of the composite films were comparatively investigated after clarifying the chemical structure of these lignin residues. This work showed a promising route to the conversion of cellulose and lignin residues into high-value composite films, and also showed chemical insights into lignin phenolic substructures for UV-blocking application.

## 2. Results and Discussion

### 2.1. Cellulose/Lignin Composite Films Preparation

**Figure 1:**
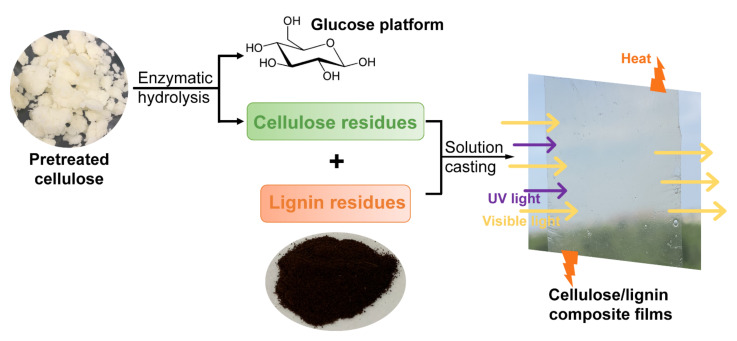
UV-blocking functional composite films fabricated using cellulose and lignin residues in a biorefinery.

Bamboo cellulose is a promising substrate for biorefinery due to its fast-growing advantage. When it was selected for bioethanol production in this work, considerable cellulose to glucose conversion of 79.2% was achieved after 12 h enzymatic hydrolysis using Cellic CTec2 (Figure 1). The remaining cellulose residue with relatively high bioconversion recalcitrance was further used for film preparation. These three assessing lignin residues, i.e., aspen and poplar lignin, and CS lignin were obtained using liquid hot water pretreatment followed by a solvent extraction method [11]. It was shown that well-defined composite films were successfully prepared using these three lignin residues with 2% or 4% lignin content. It was proposed that unlike metal oxides, which blocked UV lights through electron transitions in valence bands with the generation of radicals, lignin was suggested to block the UV light by absorbing its photon energy and further converting it to heat with the corresponding hydrophilic phenolic hydroxyl, carbonyl and carboxyl chromophores. Then, the produced heat was gradually released out of the composite films (Figure 1). To achieve a better investigation of how lignin’s chemical structure influences the overall properties of the resulting composite films, the lignin phenolic substructures’ and hydroxyl groups’ analyses were subsequently conducted.

### 2.2. Lignin Characterization Using ^31^P-NMR

**Figure 2:**
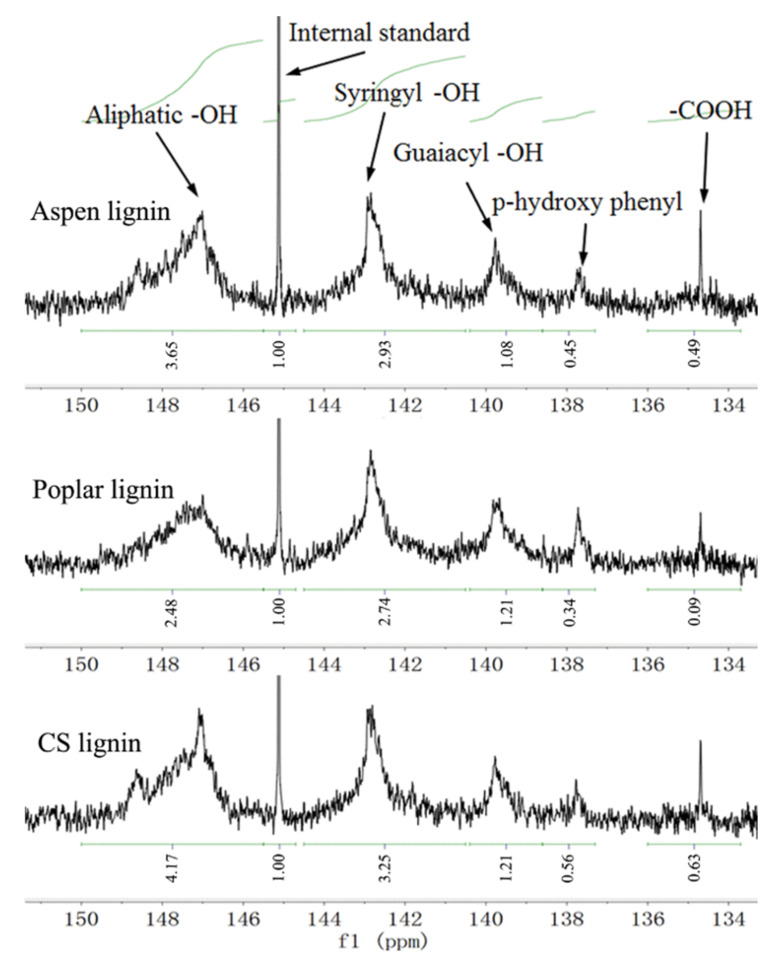
Quantitative ^31^P-NMR spectra of these three lignin residues tagged TMDP phosphorous reagent using cyclohexanol as internal standard.

These three lignin residues were characterized using the emerging quantitative ^31^P nuclear magnetic resonance (NMR) technique according to previous protocol [21]. In this procedure, lignin was phosphitylated; thus, all the hydroxyl groups including phenolic, aliphatic and carboxylic hydroxyls were phosphorus-tagged. Then, these functional groups could be readily quantified by ^31^P-NMR spectroscopy. The internal standard of cyclohexanol was added and also phosphitylated for the calculation purpose. It was shown that representative phenolic, aliphatic and carboxylic hydroxyls were all detected for all these three lignin residues (Figure 2). When the contents and locations of the hydroxyl groups in these three lignin residues were determined and compared, it was shown the total phenolic hydroxyls content was 2.73, 2.62 and 3.07 mmol g^−1^ for aspen, poplar and CS lignin, respectively (Table 1). Generally, the total phenolic hydroxyls content of lignin would significantly increase to above 3.0 mmol g^−1^ through β–O–4 cleavage at severe conditions [21,22]. However, in the work reported here, this lignin residue showed rather low phenolic hydroxyls content after liquid hot water pretreatment. This also indicated the structural recalcitrance and technical challenges of valorization of these lignin residues [11,23]. Although these three lignin residues were S–G–H in the type, CS lignin showed slightly higher content of syringyl phenolic and *p*-hydroxyphenyl hydroxyl groups, likely due to its higher extent of lignin fragmentation. CS lignin also exhibited much higher content of carboxylic acid hydroxyl groups, which was in line with the fact that herbaceous plants tend to release more carboxylic acid hydroxyl groups during lignin extraction. Both aspen and poplar are hardwood species, and their phenolic substructures were quite similar to each other. However, their aliphatic and carboxylic acid hydroxyl groups were different. Under acidic conditions, aliphatic hydroxyl groups easily underwent dehydration reaction. It appeared that poplar lignin suffered from more dehydration, thus, less content of aliphatic hydroxyl groups (1.52 mmol g^−1^) was presented. It also appeared that more severe oxidation reactions occurred for aspen lignin, and, consequently, higher content of carboxylic acid hydroxyl groups (0.30 mmol g^−1^) was observed.

### 2.3. UV-Blocking Property of Cellulose/Lignin Composite Films

**Figure 3:**
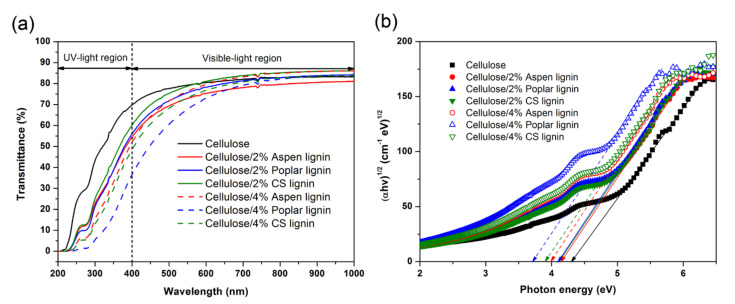
UV–Vis light transmittance spectra (**a**) and photon energy calculation (**b**) of cellulose/lignin composite films. Lignin loading was 2% and 4% for these three lignin residues.

As shown in Figure 3a, all the lignin additions could significantly enhance the UV-blocking performance of the composite films even at 2% loading. Further increasing lignin content to 4% also improved the UV-blocking property with varied extents. At lower lignin contents, these three lignin residues exhibited a very similar UV-blocking property, while at higher lignin contents, it was apparent that poplar lignin exhibited the highest UV-blocking ability, followed by CS and aspen lignin. Previous work has shown that when the same poplar lignin substrate was used for UV-blocking functional composite preparation, the UV-blocking ability was highly related to its total phenolic total phenolic hydroxyls [3,24]. However, when varied lignin residues were used in this case, no clear correlation between total phenolic hydroxyls of lignin and UV-blocking ability of the composite was observed. This showed the biomass species was also influential on the UV-blocking property, likely due to the varied chromophore groups including phenolic hydroxyls. For example, poplar lignin suffered from a higher extent of aliphatic hydroxyls dehydration, thus more carbonyl chromophores were generated. It appeared that the lignin carbonyl chromophores significantly contributed to the UV-blocking property of the composites (Table 1). A high amount of carboxylic acid hydroxyl groups (0.39 mmol g^−1^) in CS lignin actually compromised its UV-blocking performance, which was in line with our previous findings when soda/anthraquinone lignin with oxidation was used [25]. In addition, all these lignin additions had no or limited influence on the visible-light transparency of the composite films especially at 600–1000 nm wavelength. This indicated that these composite films are promising in advanced packaging with UV-blocking functionality and considerable visible-light transparency.

To obtain a better understanding of how lignins’ chemical structure influenced the overall UV-blocking performance of the composite films and also gave a quantitative analysis of these films, the optical energy band gap (Eg) was obtained by translating the UV–Visible transmittance spectra into Tauc’s plot with the frequency dependent absorption coefficient according to the previous report [19]. Photons with energy higher than the band gap energy would be absorbed by the corresponding lignin chromophores in the cellulose/lignin composite films. When the Eg values of these composite films were calculated, it was decreased from 4.31 eV for neat cellulose film to ~4.10 eV with 2% lignin loading, and further to ~3.72 eV with 4% lignin loading (Figure 3b). The decreased Eg value clearly showed that more UV lights with wider wavelength range could be blocked with lignin incorporations.

### 2.4. Mechanical Property of Cellulose/Lignin Composite Films

**Figure 4:**
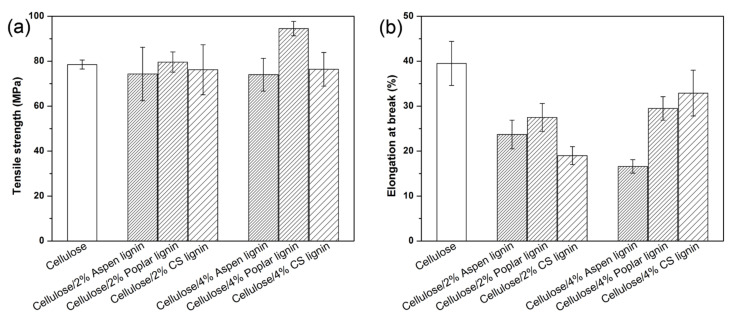
Tensile strength (**a**) and elongation at break (**b**) of neat cellulose and cellulose/lignin composite films.

As the mechanical property is a key parameter to describe a given film material, tensile strength and elongation at break were measured and comparatively analyzed (Figure 4). The tensile strength was 78.5 MPa for neat cellulose film, and 74.3~79.2 MPa for cellulose/lignin film with 2% lignin loading, and 74.0~94.5 MPa for cellulose/lignin film with 4% lignin (Figure 4a). No obvious decrease or increase in the tensile strength was observed, likely due to the amorphous nature of lignin, whereas other natural polymers such as crystalline cellulose or polydopamine that had well-defined dimensions could usually reinforce a polymer matrix [3,19]. It was interesting that the composite films with poplar lignin that showed better UV-blocking performance also exhibited higher tensile strength. This indicated that poplar lignin and the cellulose matrix had better interface bonding as the lignin fragments were a low-molecular-weight amphiphilic polymer with hydrophobic aromatics and hydrophilic hydroxyl groups. It was shown that the abundant lignin hydrophilic hydroxyl groups could interact with cellulose hydroxyl groups to form a hydrogen bonding network; therefore, cellulose macromolecular chain movement was restricted. These interactions consequently lowered the elongation at break of the composite films. As shown in Figure 4b, the elongation at break was 39.5% for neat cellulose film, but was decreased to ~19.0% and further to 16.6% after lignin addition with 2% and 4% loading, respectively. Overall, lignin addition enhanced the rigidity of the composite films without compromising their tensile strength. Since the cellulose residue with a rather lowered degree of polymerization was used for the composite films’ preparation, the tensile strength was slightly lower than those of previous reported films [17]. When representative polyethylene film was selected for comparison, the resulting cellulose/lignin composite films showed much higher tensile strengths than polyethylene films (usually 15–40 MPa).

### 2.5. Crystalline Morphology and Thermal Stability Analysis

**Figure 5:**
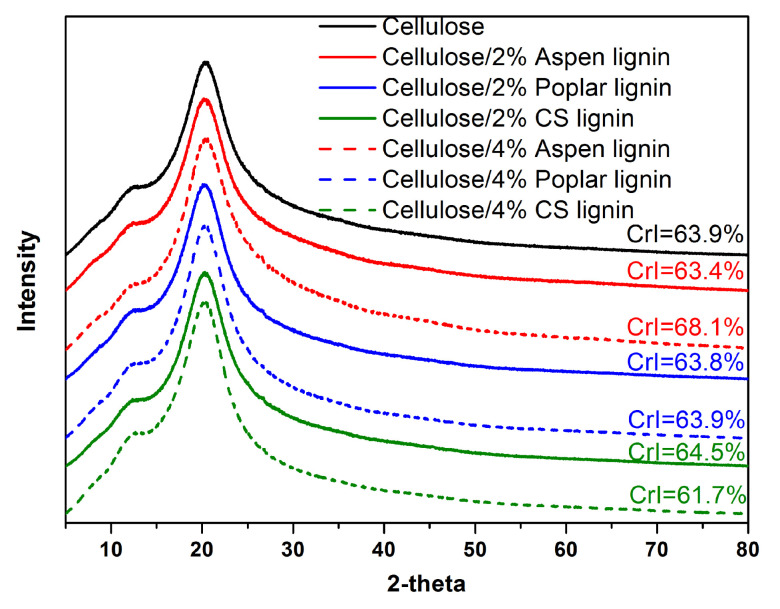
XRD patterns and crystallinity index (CrI) of neat cellulose and cellulose/lignin composite films.

To further investigate the crystalline morphology of the composite films and gain insights into the interaction between cellulose and lignin, X-ray diffraction (XRD) analysis was conducted. Generally, native plant cellulose showed a characteristic cellulose I_β_ crystalline morphology [17], which was changed to cellulose III_I_ after the dissolution–regeneration process for film casting (Figure 5). All these regenerated cellulose films showed typical strong Bragg peaks at 2θ = 12.6° and 20.4° in their XRD patterns. When the crystallinity index (CrI) was calculated, it ranged from 61.7 to 68.1%. It appeared that the influence of lignin addition on cellulose crystallization was quite complicated. As discussed before, lignin was an amphiphilic polymer with amorphous structure. The low-molecular-weight lignin fragments disordered cellulose arrangement rather than acting as the nucleating agent [3], while the abundant hydroxyl groups tended to form an additional hydrogen bonding network; thus, enhancing the cellulose crystallization process. The antagonistic and competitive interactions resulted in the unpredictable CrI of the composite films.

**Figure 6:**
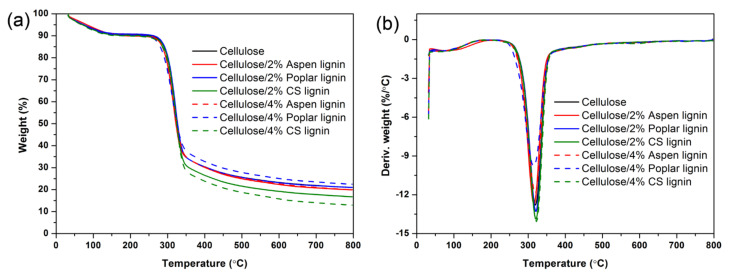
Thermogravimetric (**a**) and differential thermogravimetric (**b**) curves of neat cellulose and cellulose/lignin composite films.

Thermal stability analysis could provide useful information for the downstream applications of these composite films. Figure 6 shows the TG and DTG curves of neat cellulose and cellulose/lignin composite films under nitrogen atmosphere, while the main parameters are summarized in Table 2. A rather wide degradation temperature ranging from about 200 to 500 °C was observed for all the assessed sample films. It appeared that lignin shared a similar degradation temperature with cellulose. Typical cellulose initial decomposition, maximum decomposition and end decomposition temperatures of 296.0, 319.4 and 340.4, respectively, were observed for neat cellulose film, corresponding to a residual char of 21.0%, while the incorporation of lignin showed limited influence on those decomposition temperatures but significantly influenced the charring process. Basically, lignin was an aromatics-rich polymer; thus, it exhibited a rather high char residual after decomposition [3]. However, during the pretreatment or extraction process, quite a large amount of oxygen-containing functional groups such as carboxylic hydroxyl groups were introduced. This significantly alleviated the charring process. As shown in Table 2, the composite films with CS lignin indeed showed lowered char residue, which was 16.8% and 13.0% with 2% and 4% CS lignin incorporation, respectively. Earlier work has shown that when lignin was incorporated into polyvinyl alcohol or xylan matrix that had a rather low initial decomposition temperature, it enhanced the overall composite stability through capture of the free radicals generated during the pyrolysis process [25]. In this work, it appeared that this retarding effect was not clearly shown due to the already considerably high initial decomposition temperature of cellulose, while for poplar lignin that had considerable aromatics and low carboxylic hydroxyl groups, it actually facilitated the charring process. It appears that the incorporation of these lignin residues into cellulose film would not compromise its thermal stability.

As the emerging cellulose nanofibrils/lignin nanoparticles films also employed cellulose and lignin as the starting materials, their overall properties were consequently compared with prevalent reported ones [26,27,28,29]. It was shown that the visible-light transparency of cellulose/lignin composite films was higher than those cellulose nanofibrils/lignin nanoparticles films due to the employed dissolution and regeneration process. During the film preparation process for cellulose nanofibrils, the three-dimensional fibrillar structure with a high length to diameter ratio of cellulose still remained; therefore, the mechanical strength and crystalline index of those nanocomposite films were higher. Both the lignin nanoparticles and lignin fragments were well dispersed in the film preparation system, and the resulting nanocomposite films and cellulose/lignin films reported in this work both showed considerable UV-blocking property. This comparative analysis indicated that the composite films prepared in this work could share similar applications fields with the emerging cellulose nanofibrils/lignin nanoparticles films.

## 3. Materials and Methods

### 3.1. Materials

Aspen, poplar and corn stover substrates used for lignin extraction were obtained from a local industry in Chengdu, Sichuan province, China. Bleached Kraft bamboo pulp was kindly donated by a local paper industry (Chengdu, China). It had a cellulose content of 78.3% and xylan content of 15.5%. Cyclohexanol and 2-chloro-4,4,5,5-tetramethyl-1,3,2-dioxaphospholane (TMDP, Product No. 447536) were purchased from Sigma-Aldrich (Shanghai, China). Cellic CTec2 enzyme was kindly provided by Novozymes in Beijing of China (protein concentration was 228.7 mg mL^−1^, enzyme activity was 144 FPU mL^−1^). Other reagents and solvents were purchased from Kelong Chemical Regent Co., Ltd. (Chengdu, China) and used as received.

### 3.2. Cellulose and Lignin Residue Acquirement

Cellulose residue was acquired through enzymatic hydrolysis after glucose production. Enzymatic hydrolysis was carried out at 10% (*w/v*) solids loading in sodium acetate buffer (50 mM, pH 4.8), 50 °C, 150 rpm in an incubator with an enzyme loading (Cellic CTec2) of 20 mg g^−1^ cellulose. After 12 h of hydrolysis, 0.5 mL of the hydrolysate was taken and incubated on a hot plate at 100 °C for 10 min to deactivate the enzyme. The sample was subsequently centrifuged and the supernatant collected and analyzed for glucose conversion using HPLC [24]. The cellulose residue was obtained by filtration and washed for further use.

Lignin residue was acquired through liquid hot water pretreatment followed by mild 1,4-dioxane extraction. Liquid hot water extraction was conducted using a 1.5 L autoclave reactor (GCF-1.5, Dalian Autocontrol Equipment Co., Dalian, China). In total, 30 g (dry biomass) of substrate was soaked in water overnight at a liquid to solid ratio of 30:1 [23]. Then, it was cooked in the autoclave reactor at 170 °C for 40 min with a stirring speed of 150 r min^−1^. To keep the original chemical structure of lignin after liquid hot water pretreatment as much as possible, the obtained substrate without drying was extracted with 1,4-dioxane/water (90/10, *v*/*v*) system at 100 °C for 24 h. Lignin was obtained by vacuum distillation to remove 1,4-dioxane and water. Then, lignin was washed and dried in an oven for further use.

### 3.3. Cellulose/Lignin Composite Film Preparation

The 8% (*w*/*w*) DMAc/LiCl solvent system used for film casting was prepared according to previous work [17]. The resulting cellulose residue was activated by methanol and DMAc from the previous procedure before dissolution. Basically, cellulose residue was obtained by vacuum filtration and then dispersed in water. Solvent exchange with methanol was conducted to remove residual water. Then, solvent exchange with DMAc was conducted to remove methanol. The activated cellulose residue was added to the above 8% (*w*/*w*) DMAc/LiCl solvent system with string until a transparent solution was obtained. Then, 2% or 4% (based on cellulose weight) lignin sample was added into cellulose solution. The resulting mixture was cast on a glass plate and kept at ambient conditions overnight to form a gel. To remove DMAc and LiCl traces, the gel-like film was washed thoroughly with distilled water for 24 h. Then, the obtained cellulose/lignin composite film was dried in a vacuum oven. The thickness of the obtained composite films was about 20 μm.

### 3.4. UV-Blocking Assessment

The UV-blocking property of cellulose/lignin composite films was tested on a UV–Visible spectrophotometer (U-2910, HITACHI, Tokyo, Japan). The spectra was obtained at a scanning step of 2 nm from 200 to 1000 nm. The Eg of the composite film was calculated by Tauc’s expression. Detailed calculation process could be found in our previous work [3].

### 3.5. Characterization

^31^P-NMR analysis was conducted to quantify the content and location of lignin hydroxyl groups [25]. An amount of 20 mg, accurately weighed, of lignin was dissolved in 500 μL anhydrous pyridine and deuterated chloroform mixture (1.6:1, *v*/*v*) with stirring. Then, 100 μL of cyclohexanol (10.85 mg mL^−1^ in anhydrous pyridine and deuterated chloroform 1.6:1, *v*/*v*) and 100 μL of chromium (III) acetylacetonate solution (5 mg mL^−1^ in anhydrous pyridine and deuterated chloroform 1.6:1, *v*/*v*) were further added as an internal standard and relaxation reagent, respectively. The mixture was reacted with 100 μL of phosphitylating reagent TMDP and transferred into a 5 mm NMR tube. The NMR spectra of freshly prepared samples were acquired immediately at room temperature on a 600 MHz spectrometer (Bruker, Fällanden, Switzerland) equipped with a QNP cryoprobe. Chemical shifts were calibrated relative to the phosphitylation product of TMDP with water (sample moisture), which gave a sharp and stable signal at 132.2 ppm.

Tensile strength and elongation at break were measured according to standard method ASTM D882-12 on Instron 5567 Universal Testing Machine (Instron, Norwood, MA, USA). Film samples were cut into dimensions of 50 × 10 mm and five specimens of each sample were tested.

X-ray diffraction (XRD) testing was conducted on a Philips Analytical X’Pert X-diffractometer (Philips Co., Amsterdam, The Netherlands), using Cu–Ka radiation (λ = 0.1540 nm) at an accelerating voltage of 40 kV and the current of 40 mA. The data were collected from 2θ = 5–80° with a step interval of 0.03°. The crystallinity index was calculated according to previous report [17].

Thermal stability was measured on a TG209 F1 instrument (NETZSCH Co., Selb, Germany). About 5–8 mg of each composite film was heated in a platinum crucible from room temperature to 800 °C at a heating rate of 20 °C min^−1^ under N_2_ atmosphere.

## 4. Conclusions

Cellulose/composite films with UV-blocking functionality could be prepared using cellulose and lignin residues through a dissolution–regeneration process. Unlike the prevalent disposal method where cellulose and lignin residues were usually burned for heat generation, this technique showed the high promise to valorize those residues. This conversion process could be facilely integrated into current bioethanol-based integrated biorefineries where cellulose and lignin residues are produced in large amounts. The incorporation of lignin into the composite films enhanced the UV-blocking property without compromising their tensile strength and thermal stability. The UV-blocking ability of the composite films was highly dependent on the lignin species and contents. Poplar lignin had a considerable content of chromophores and showed the best UV-blocking enhancement among these three assessing lignins. This work showed a facile way to valorize recalcitrant cellulose and lignin residues through composite films fabrication, and also provided useful information on UV-blocking additive preparation. The prepared cellulose/lignin composite film was promising in biodegradable advanced packaging. This work also showed lignin residues could be used as metallic oxide alternatives for UV-blocking additive application.

## Figures and Tables

**Table 1:** Contents and locations of hydroxyl groups in these three lignin residues quantified by ^31^P-NMR technique.

-OH Content (mmol g^−1^)	Aspen Lignin	Poplar Lignin	CS Lignin
Aliphatic -OH	2.23	1.52	2.55
Syringyl phenolic -OH	1.79	1.68	1.99
Guaiacyl phenolic -OH	0.66	0.74	0.74
*p*-hydroxyphenyl -OH	0.28	0.21	0.34
Carboxylic acid -OH	0.30	0.06	0.39
Total phenolic -OH	2.73	2.62	3.07

**Table 2:** Thermal parameters of these cellulose/lignin composite films that were calculated from TG and DTG analysis.

Sample	T_onset_ (°C)	T_max_ (°C)	T_comp_ (°C)	Char (%)
Cellulose	296.0	319.4	340.4	21.0
Cellulose/2% Aspen lignin	292.6	315.7	337.5	19.9
Cellulose/2% Poplar lignin	297.9	321.0	340.8	21.0
Cellulose/2% CS lignin	297.8	320.7	340.9	16.8
Cellulose/4% Aspen lignin	292.8	318.8	341.7	19.9
Cellulose/4% Poplar lignin	285.0	315.0	341.7	22.5
Cellulose/4% CS lignin	297.9	322.1	341.7	13.0

## Data Availability

Not applicable.

## Sample Availability

Samples of the lignin and composite films are available from the authors upon reasonable request.
